# Effect of Double Bond Position on 2-Phenyl-benzofuran Antioxidants: A Comparative Study of Moracin C and *Iso*-Moracin C

**DOI:** 10.3390/molecules23040754

**Published:** 2018-03-24

**Authors:** Xican Li, Hong Xie, Ruicai Zhan, Dongfeng Chen

**Affiliations:** 1 School of Chinese Herbal Medicine, Guangzhou University of Chinese Medicine, Waihuan East Road No. 232, Guangzhou Higher Education Mega Center, Guangzhou 510006, China; xiehongxh1@163.com (H.X.); michael_zcx@hotmail.com (R.Z.); 2 Innovative Research & Development Laboratory of TCM, Guangzhou University of Chinese Medicine, Waihuan East Road No. 232, Guangzhou Higher Education Mega Center, Guangzhou 510006, China;; 3 School of Basic Medical Science, Guangzhou University of Chinese Medicine, Waihuan East Road No. 232, Guangzhou Higher Education Mega Center, Guangzhou 510006, China;; 4 The Research Center of Basic Integrative Medicine, Guangzhou University of Chinese Medicine, Waihuan East Road No. 232, Guangzhou Higher Education Mega Center, Guangzhou 510006, China

**Keywords:** 2-phenyl-benzofuran, antioxidant, moracin, double bond, positional isomeric effect

## Abstract

Two 2-phenyl-benzofurans, moracin C {2-[3′,5′-dihydroxy-4′-(3-methlbut-2-enyl)phenyl]-6-hydroxybenzofuran} and its isomer *iso-*moracin C{2-[3′,5′-dihydroxy-4′-(3-methlbut-1-enyl)phenyl]-6-hydroxybenzofuran}, were comparatively studied using redox-related antioxidant assays and non-redox antioxidant assays. Moracin C always resulted in higher IC_50_ values than *iso*-moracin C in the redox-related antioxidant assays, including •O_2_^−^-inhibition, Cu^2+^-reducing power, DPPH•-inhibition, and ABTS^+^•-inhibition assays. In the non-redox antioxidant assay, moracin C and *iso*-moracin C underwent similar radical-adduct-formation (RAF), evidenced by the peaks at *m/z* 704 and *m/z* 618 in HPLC-MS spectra. In conclusion, both moracin C and *iso*-moracin C can act as 2-phenyl-benzofuran antioxidants; their antioxidant mechanisms may include redox-related ET and H^+^-transfer, and non-redox RAF. A double bond at the conjugation position can enhance the redox-related antioxidant potential, but hardly affects the RAF potential.

## 1. Introduction

**Figure 1:**
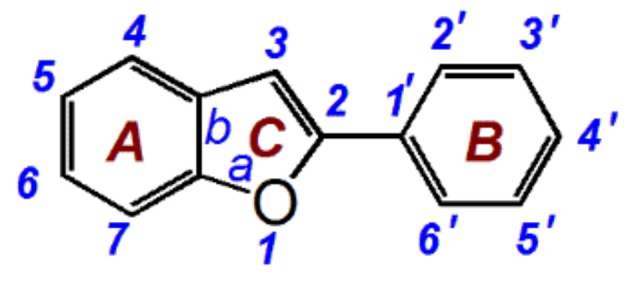
The scaffold of 2-phenyl-benzofurans.

Despite its 60-year history, naturally occurring 2-phenyl-benzofuran is not a well-known type of stilbene. In 1958, a new product was discovered in yeast and identified as **2**-(6-hydroxy-2-methoxy-3,4-methylenedioxy**phenyl**)-**benzofuran** by Meisinger et al. [1]. Since then, over sixty other 2-phenyl-benzofuran derivatives [2] have been successfully isolated from different plants, especially from *Morus alba* [3,4], *Artocarpus champeden* [5], *Erythrina addisoniae* [6], and *Calpocalyx dinklagei* [7]. Structurally, all these compounds contain a scaffold of 2-phenyl substituted benzo[*b*]furan-fused-ring (Figure 1). This scaffold is usually called 2-phenyl-benzofuran, although somestudies also refer to it as “2-arylbenzofuran” or “aryl benzofuran” [6,7,8]. The latter two terms, however, are ambiguous because aryl can refer to any heterocycle—not specifically the phenyl ring. For example, recently synthesized 2-quinolyl benzofuran derivatives are aryl benzofurans [9], but not phenyl-benzofurans. More importantly, the nomenclature 2-phenyl-benzofuran conforms with the IUPAC rule, and is identical with the name of the first isolated natural benzofuran compound [1] and a wide range of similar compounds in the literature [2]. Possibly due to the mess of terminology, some handbooks have not recorded these derivatives as an independent type of natural product. Thus, very few people have recognized natural 2-phenyl-benzofuran derivatives.

**Figure 2:**
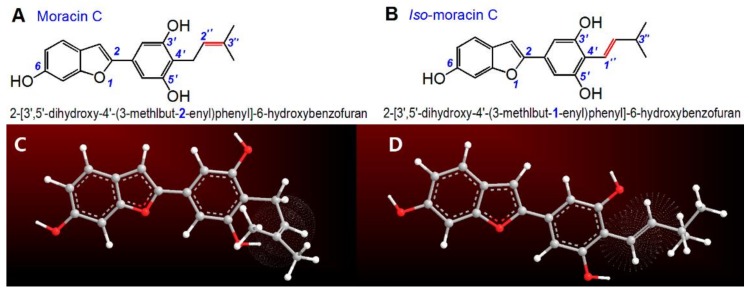
Structures and preferential conformation-based ball-stick models of moracin C and its isomer: (**A**) the structure of moracin C; (**B**) the structure of *iso*-moracin C; (**C**) the preferential conformation-based ball-stick model of moracin C; (**D**) the preferential conformation-based ball-stick model of *iso-*moracin C. The ball-stick models were created in Chem3D Pro 14.0. The three-dimensional perspective animations are shown in Video S1 and S2.

Moracin C from *Morus alba* or *Artocarpus heterophyllus* is one of the more well-known natural 2-phenyl-benzofuran derivatives [10]. As shown in Figure 2A, moracin C contains three phenolic –OH groups at the 6,3′,5′-positions. Thus, it can also be regarded as a phytophenol [11]. Of course, it is dissimilar to any of the common phytophenols, such as flavonoid [12], flavonoid glucoside [13], biflavonoid [14], volatile phenol [15], phenolic cumarin [16], phenolic alkaloid [17], phenolic acid [18,19], and phenolic acid ester [20]. Like most phytophenols, however, the characteristic phenolic moiety of moracin C makes it of interest to many researchers. Recently, Yao et al. used a cellular model to explore its inhibitory effect on the nitric oxide production of RAW264.7 cells [10]; while Zelová et al. reported its anti-inflammatory activity [21]. In addition, moracin C has also been found to inhibit fatty acid synthesis [22] and lipoxygenase levels [23], both of which are positively correlated with oxidative stress [24,25]. These three inhibitory effects of moracin C are thought to originate from an antioxidant action. However, to the best of our knowledge, there is no relevant study to date on the antioxidant action of moracin C. 

Notably, an isomer of moracin C (Figure 2B) has also been found in *Artocarpus* family [8,23]. Similar to moracin C, the isomer has been given two nonstandard names. Some published works refer it as artoindonesianin B-1 [8]. This name is readily associated with artoindonesianin B, a flavonoid from *Artocarpus champeden* (Figure S1) [5]. As seen in Figure S1, however, the flavonoid artoindonesianin B is quite different from the isomer of moracin C (Figure 2B). The supplier also calls this compound 5-(6-hydroxybenzofuran-2-yl)-2-(3-methylbut-1-enyl)benzene-1,3-diol. While this name can be used to deduce its chemical structure, it does not comply with IUPAC terminology; i.e., the numbering does not start from the *O*-atom of the furan ring. This nomenclature is also different from that of the first reported natural 2-phenyl-benzofuran derivative [i.e., **2**-(6-hydroxy-2-methoxy-3,4-methylenedioxy**phenyl**)-**benzofuran**] [1] and other analogous derivatives such as **2**-[2′,4′-dihydroxy-3′-(3-methlbut-2-enyl)phenyl]-6-hydroxy**benzofuran**, **2**-[2′-methoxy-4′-hydroxy-5′-(3-methlbut-2-enyl)phenyl]-6-hydroxy**benzofuran**, and **2**-(2′-methoxy-4′-hydroxy**phenyl**)-5-(3-methlbut-2-enyl)-6-hydroxy**benzofuran** [2].

In this work, we have re-named the isomer **2**-[3′,5′-dihydroxy-4′-(3-methlbut-*1*-enyl)phenyl]-6-hydroxy**benzofuran**, according to the IUPAC rule. For convenience, we also refer to it as ***iso*-moracin C**, since it is actually the isomer of moracin C. Correspondingly, we have also assigned the IUPAC name for moracin C: **2**-[3′,5′-dihydroxy-4′-(3-methlbut-*2*-enyl)phenyl]-6-hydroxy**benzofuran**. 

As seen in**Figure 2**, the difference between moracin C and *iso*-moracin C is only the position of the C=C double bond. In moracin C, the double bond is at the 2″-position, whilst in *iso*-moracin C the double bond is at the 1″-position. At the 1″-position, the double bond facilitates conjugation with the phenyl group, and further, with the benzofuran-fused-ring. On the other hand, the double bond at the 2″-position cannot conjugate with the phenyl group or the benzofuran fused ring; it remains an isolated functional group. In other words, the difference between the isomers results in different degrees of conjugation. This change is obviously dissimilar to that observed in other isomers such as ferulic acid, isoferulic acid [26], and atractylenolides [27]. Conjugation, in turn, can change the electron distribution within the molecule, significantly affecting the antioxidant properties of the phytophenol [28]. In this work, therefore, we postulate that the double bond positional isomerization between moracin C and *iso*-moracin C will affect their antioxidant abilities. The present study uses spectrophotometry and HPLC-MS chemical approaches to comparatively explore the antioxidant action of moracin C and its isomer. Spectrophotometry is used to evaluate the superoxide radical (•O_2_^−^)-inhibition, Cu^2+^-reducing power, DPPH•-inhibition, and ABTS^+^•-inhibition; while HPLC-MS is used to measure the RAF potential. Our study will help us to understand the antioxidant ability and mechanisms of moracin C and *iso*-moracin C. More importantly, it will also provide new information about the effect of the double bond position on the antioxidant properties of phytophenols—especially phenolic 2-phenyl-benzofurans.

## 2. Results and Discussion

Excessive reactive oxygen species (ROS) or reactive nitrogen species (RNS) are the source of cellular oxidative stress; decreasing either can effectively relieve the oxidative stress in the cells. The process by which this occurs is commonly called “antioxidation”; essentially, this process consists of reactions which inhibit ROS or RNS accumulation. •O_2_^−^ is a significant form of ROS. In the present study, it was inhibited by both moracin C and *iso*-moracin C in a dose-dependent manner (Figure S2). Furthermore, both moracin C and *iso*-moracin C displayed higher percentages of •O_2_^−^ inhibition compared to Trolox, a standard antioxidant. This indicated that both moracin C and *iso*-moracin C are good antioxidants. 

ROS and RNS induce cellular oxidative stress and injure biomolecules in cells due to the presence of unpaired electrons in their structures which make them highly unstable. However, as long as the unpaired electron is paired by an external electron donating system, these species can be inactivated and become harmless to cells. As such, electron-transfer (ET) from antioxidants (specifically phenolic antioxidants) to ROS or RNS is proposed as an important antioxidant approach [29], and antioxidants are frequently investigated for their ET potential using chemical approaches. 

From the perspective of chemistry, ET is actually a redox reaction. For example, the metal ion Cu^2+^ can be reduced to Cu^+^ by gaining an electron from another molecule. Accordingly, the Cu^2+^-reducing capacity assay can experimentally characterize the ET potential of an antioxidant. This assay is called the cupric ion reducing antioxidant capacity (CUPRAC) assay and is carried out under physiological pH 7.4 [30]. In the present study, both moracin C and *iso*-moracin C exhibited relative Cu^2+^ reducing power which was dependent on the dosage (Figure S3), indicating that both possess the ET potential under physiological conditions.

However, ET from the phenolic moiety is always accompanied by proton (H^+^) transfer. A typical application instance is the quinhydrone electrode, which has already been used for determination of pH values in analytical chemistry. The principle of the electrode is the synergism between ET and H^+^-transfer. Such synergism can also occur in biological systems. For instance, ubiquinone in cellular mitochondria can reversibly accept electron and proton via the hydroquinone form, i.e., ubiquinol with phenolic moiety [31]. Thus, it is postulated that H^+^-transfer at the phenolic moieties of moracin C and *iso*-moracin C may also accompany their ET process. This postulation is supported by the experimental results from the ABTS•^+^-inhibition and DPPH•-inhibition assays. Previous studies suggested that both of these antioxidant assays involve both ET and H^+^-transfer. Specifically, their antioxidant mechanisms can be described as loss single electron-transfer (SPLET) [12,32], proton coupled electron-transfer (PCET) [33,34], sequential electron H^+^-transfer (SEPT) [14,33,35], and even hydrogen atom transfer (HAT) [14,15,36]. HAT, however can be considered as a process where ET and H^+^ are transferred inseparably. In fact, even the ET-based CUPRAC assay could liberate H^+^ when it is used for phenolic antioxidants [37]; the aforementioned •O_2_^−^-inhibition assay by phenolic antioxidants has also recently been reported to comprise ET and H^+^-transfer [38,39]. 

**Figure 3:**
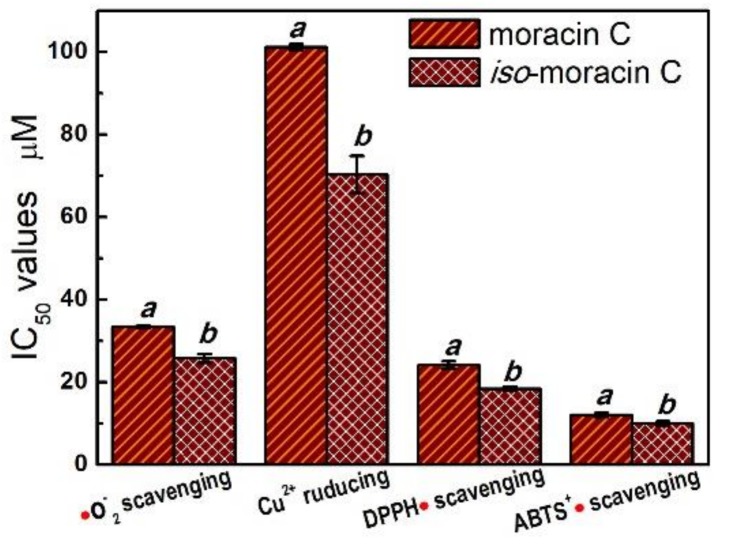
The IC_50_ values of moracin C and *iso*-moracin C in antioxidant assays, including •O_2_^−^-inhibition assay, CUPRAC assay, DPPH•-inhibition assay, and ABTS^+^•-inhibition assay.

In short, as phenolic 2-phenyl-benzofurans, moracin C and *iso*-moracin C may likely exhibit the antioxidant effect through redox-related mechanisms, which are characterized by ET and H^+^-transfer. However, as shown in Figure 3, moracin C and *iso*-moracin C showed significantly different antioxidant levels in •O_2_^−^-inhibition, CUPRAC, DPPH•-inhibition, and ABTS^+^•-inhibition assays. In the four antioxidant assays, *iso*-moracin C always gave lower IC_50_ values than moracin C, implying that *iso*-moracin C is more active than moracin C in the redox-related reactions. Structurally, the sole difference between two isomers is the double bond (C=C) position. Therefore, their different redox-related antioxidant potentials can only be attributed to the double bond position. 

The IC_50_ value was defined as the final concentration of 50% radical inhibition or relative reducing power and calculated by linear regression analysis and expressed as the mean ± SD (*n* = 3). The linear regression was analyzed by Origin 6.0 professional software. The IC_50_ value (μg/mL) was converted into μM and collected in brackets. The IC_50_ value in μM with different superscripts (a or b) in the same row are significantly different (*p* < 0.05). The dose–response curves of the positive control Trolox are listed in Figure S2–S5. 

As mentioned above, in *iso*-moracin C molecule, 1″-C=C bond conjugates with the 2-phenyl-benzofuran core to extend the π-π system. Density functional response theory has indicated that the extended π-π conjugation has a stronger capacity to stabilize the radical species via delocalization of the π-electrons [40]. Hence, *iso*-moracin C molecule, with an extended π-π conjugation, exhibited stronger redox-related antioxidant potential than moracin C. It can be inferred that, if double bond position can extend the π-π conjugative system, it can correspondently enhance the redox-related antioxidant potential. 

It must be emphasized that besides the redox-related pathways, some non-redox reactions may also occur during the antioxidant process. A typical reaction is the radical-adduct-formation (RAF) reaction. In fact, RAF was observed in DPPH•-inhibition [16], •O_2_^−^-inhibition [41], and ABTS^+^•-inhibition can [42]. In order to verify the possibility of the RAF pathway by moracin C and *iso*-moracin C, each of them was mixed with the DPPH• radical and the reaction products analyzed using HPLC-MS. 

**Figure 4:**
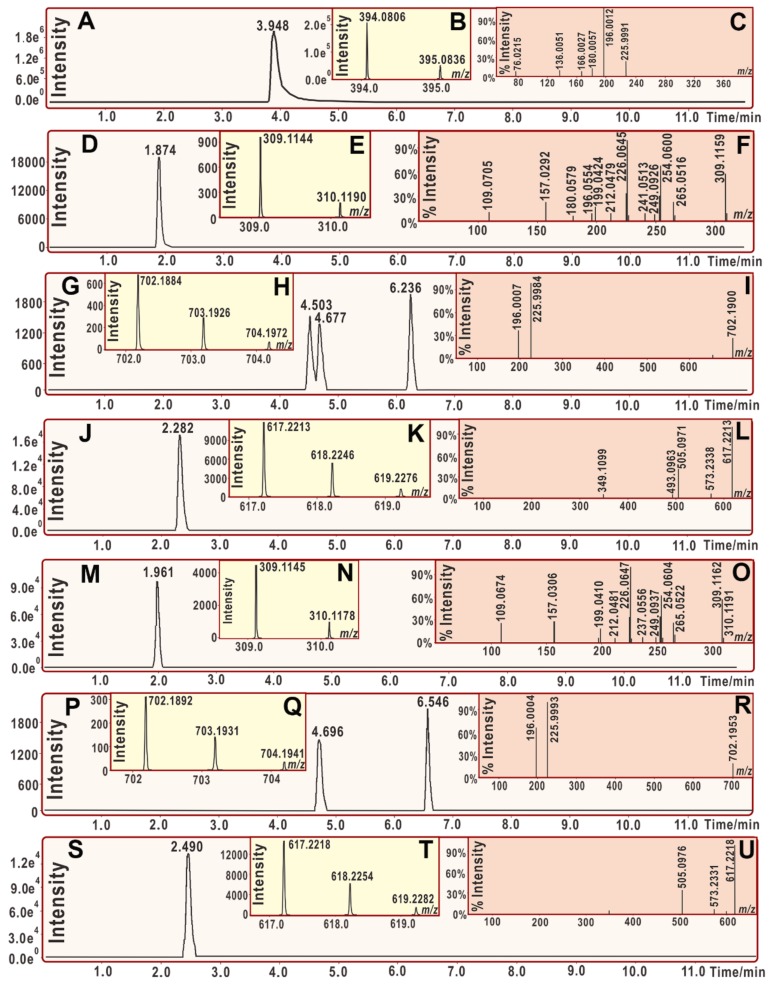
The main results of UPLC-MS analysis:(**A**, Chromatogram of DPPH• when the formula C_18_H_12_N_5_O_6_ was extracted; **B**, Primary MS spectra of DPPH•; **C**, Secondary MS spectra of DPPH•; **D**, Chromatogram of moracin C when the formula [C_19_H_18_O_4_-H]^−^ was extracted; **E**, Primary MS spectra of moracin C; **F**, Secondary MS spectra of moracin C; **G**, chromatogram of RAF product of moracin C-DPPH when the formula [C_37_H_29_N_5_O_10_-H]^−^ was extracted; **H**, primary MS spectra of RAF product of moracin C-DPPH; **I**, secondary MS spectra of RAF product of moracin C-DPPH; **J**, chromatogram of RAF product of moracin C-moracin C when the formula [C_38_H_34_O_8_-H]^−^ was extracted; **K**, primary MS spectra of RAF product of moracin C-moracin C; **L**, secondary MS spectra of RAF product of moracin C-moracin C.; **M**, Chromatogram of *iso*-moracin C when the formula [C_19_H_18_O_4_-H]^−^ was extracted; **N**, Primary MS spectra of *iso*-moracin C; **O**, secondary MS spectra of *iso*-moracin C; **P**, chromatogram of RAF product of *iso*-moracin C-DPPH when the formula [C_37_H_29_N_5_O_10_-H]^−^ was extracted; **Q**, primary MS spectra of RAF product of *iso*-moracin C-DPPH; **R**, secondary MS spectra of RAF product of *iso*-moracin C-DPPH; **S**, chromatogram of RAF product of *iso*-moracin C-*iso*-moracin C when the formula [C_38_H_34_O_8_-H]^−^ was extracted; **T**, primary MS spectra of RAF product of *iso*-moracin C-*iso*-moracin C; **U**, secondary MS spectra of RAF product of *iso*-moracin C-*iso*-moracin C.

As seen in Figure 4A–F, DPPH• standard yielded molecular ion peaks (*m*/*z* 394–394), and two fragments (*m*/*z* 196 and 226); while moracin C standard produced molecular ion peaks (*m*/*z* 309–310). The product mixture of moracin C with DPPH• gave molecular ion peaks (*m*/*z* 702–703) (Figure 4G–H). The RAF product moracin C-DPPH might be further broken to give *m*/*z* 196 and 226 fragments (Figure 4I). The molecular ion peaks (*m*/*z* 702–703) and fragment peaks (*m*/*z* 196 and 226) strongly indicated a RAF product moracin C-DPPH. In addition, the product mixture of moracin C with DPPH• also gave the peaks of *m*/*z* 617–618 (Figure 4K). These are regarded as the dimeric moracin C-moracin C. Now it is clear that, when mixed with DPPH•, moracin C can bring about two RAF products, i.e., moracin C-DPPH and moracin C-moracin C. 

Similarly, *iso*-moracin C also produced MS peaks at *m/z* 702–703 and *m/z* 617–618 (Figure 4Q,T), indicating the generation of *iso-*moracin C-DPPH and dimeric *iso-*moracin C-*iso-*moracin C. Further analysis revealed that the secondary MS spectra of *iso-*moracin C-DPPH highly resembled those of moracin C-DPPH (Figure 4I,R). It can be deduced that, DPPH moiety linked to the ring scaffold not side-chain. This is because if DPPH moiety linked to the side-chain containing C=C, the different positions of C=C should cause different MS fragments between moracin C-DPPH and *iso-*moracin C-DPPH. On the other hand, the similarity between moracin C-DPPH and *iso-*moracin C-DPPH further indicated that, the aforementioned double bond (C=C) position had actually no effect towards RAF products. 

In a word, both moracin C and *iso-*moracin C may similarly exert their antioxidant action via a RAF pathway. Through the RAF pathway, they can be transformed into stable dimers or adducts with radical-containing reactants. The C=C position is thought to have negligible effect on the RAF pathway. 

## 3. Materials and Methods 

### 3.1. Chemicals 

Moracin C (CAS 69120-06-5, C_19_H_18_O_4_, M.W. 310.4, purity 97%, faint yellow, Figure S6) and *iso*-moracin C (CAS936006-11-0, C_19_H_18_O_4_, M.W. 310.4, purity 97%, yellow, Figure S6) were obtained from BioBioPha Co., Ltd. (Kunming, China). 1,1-Diphenyl-2-picryl-hydrazyl radical (DPPH•), (±)-6-hydroxyl-2,5,7,8-tetramethylchromane-2-carboxylic acid (Trolox), pyrogallol, and 2,9-dimethyl-1,10-phenanthroline (neocuproine) were purchased from Sigma-Aldrich Shanghai Trading Co. (Shanghai, China). (NH_4_)2ABTS [2,2′-azino-bis(3-ethylbenzo-thiazoline-6-sulfonic acid diammonium salt)] was obtained from Amresco Chemical Co. (Solon, OH, USA).Acetonitrile was of HPLC grade and formic acid was of LC-MS grade. Other reagents were of analytical grade. 

### 3.2. Superoxide Anion (•O_2_^−^) Inhibiting Assay (Spectrophotometry)

Superoxide anion (•O_2_^−^) inhibiting activity was measured using a pyrogallol autooxidation method that was previously improved in our laboratory [43]. Briefly, the sample was dissolved in methanol at 1 mg/mL. The sample solution (*x* = 2–10 μL) was mixed with Tris-HCl buffer (980-*x* μL, 0.05 M, pH 7.4) containing EDTA (1 mM). After 20 μL pyrogallol (60 mM in 1 mM HCl) was added, the mixture was vigorously shaken at room temperature. The absorbance of the mixture was measured (Unico 2100, Shanghai, China) at 325 nm every 30 s for 5 min. Tris-HCl buffer was used as a blank. The •O_2_^−^ inhibiting ability was calculated as follows:

$$\mathrm{Inhibition}\%=\frac{\left(\frac{\Delta A_{325nm,\mathrm{control}}}{T}\right)-\left(\frac{\Delta A_{325nm}{}_{,\mathrm{sample}}}{T}\right)}{\left(\frac{\Delta A_{325nm}{}_{,\mathrm{control}}}{T}\right)}\times 100\%$$

Here, Δ*A_325nm_,_control_* is the increment in the absorbance at 325 nm (*A_325nm_*) of the mixture without the sample, and Δ*A_325nm_,_sample_* is the increment in *A_325nm_* of the mixture with the sample; *T* = 5 min.

### 3.3. CUPRAC Assay (Spectrophotometry) 

Cupric ion reducing antioxidant capacity (CUPRAC) assay was determined based on the method proposed by Apak et al. [44], with small modifications as presented in the literature of Jiang [45]. Twelve μL CuSO_4_ solution (0.01 M) and 12 μL ethanolic neocuproine solution (7.5 × 10^−^^3^ M) were added to a 96-well and mixed with different concentrations of samples (10–50 μg/mL). The total volume was then adjusted to 100 μL with a CH_3_COONH_4_ buffer solution (0.1 M), and mixed again to homogenize the solution. The mixture was maintained at room temperature for 30 min, and the absorbance was measured at 450 nm on a microplate reader (Multiskan FC, Thermo Scientific, Shanghai, China). The relative reducing power of the sample was calculated using the formula:

$$\mathrm{Relative}\;\mathrm{reducing}\;\mathrm{effect}\%=\frac{A-A_{\min}}{A_{\max}-A_{\min}}\times 100\%$$

where *A* is the absorbance of sample at 450 nm, *A*_max_ is the maximum absorbance at 450 nm, and *A*_min_ is the minimum absorbance in the test at 450 nm.

### 3.4. DPPH•-Scavenging and ABTS•^+^-Scavenging Assays (Spectrophotometry)

DPPH• radical scavenging activity was determined as previously described [46]. Briefly, 80 μL of DPPH• solution (0.1 mol/L) was mixed with methanolic sample solutions having the indicated concentration (0.125 mg/mL, 1–5 μL). The mixture was maintained at room temperature for 30 min, and the absorbance was measured at 519 nm on a microplate reader. The percentage of DPPH• scavenging activity was calculated using the following equation:

$$\mathrm{Scavenging}\;\%\;=\;\frac{A_{0}-A}{A_{0}}\;\times \;100\%$$

where *A*_0_ indicates the absorbance of the blank at the specified wavelength and *A* indicates the absorbance of the sample at the specified wavelength.

The ABTS•^+^- scavenging activity was evaluated according to the method described by Wang et al. [47]. The ABTS•^+^ was produced by mixing 0.2 mL of (NH_4_)_2_ABTS (7.4 mmol/L) with 0.35 mL of potassium persulfate (2.6 mmol/L). The mixture was kept in the dark at room temperature for 12 h to allow completion of radical generation, and then diluted with distilled water (~1:20), such that its absorbance at 734 nm was measured on a microplate reader. To determine the scavenging activity, the test sample (*x* = 1–5 μL, 0.125 mg/mL) was added to (20 − *x*) μL of distilled water followed by 80 μL of ABTS•^+^ reagent, and the absorbance at 734 nm was measured 3 min after the initial mixing, using distilled water as the blank. The percentage inhibition of the samples was calculated based on the equation governing DPPH• scavenging. 

### 3.5. Determining DPPH• Reaction Products with Moracin C or Iso-Moracin C (UPLC-ESI-Q-TOF-MS/MS Analysis) 

The reaction conditions were based on the maclurin experiment [33]. In brief, a methanolic solution of moracin C was mixed with a methanolic solution of DPPH• at a molar ratio of 1:2, and the resulting mixture was incubated for 5 h at room temperature. The product was then filtered through a 0.22-μm filter for UPLC-ESI-Q-TOF-MS/MS analysis.

The UPLC-ESI-Q-TOF-MS/MS analysis was based on our reported method [48]. The UPLC-ESI-Q-TOF-MS/MS analysis system was equipped with a C_18_ column (2.0 mm i.d. × 100 mm, 2.2 μm, Shimadzu Co., Kyoto, Japan). The mobile phase used for the elution of the system consisted of a mixture of acetonitrile (phase A) and 0.1% aqueous formic acid (phase B). The column was eluted at a flow rate of 0.2 mL/min with the following gradient elution program: 0–2 min, maintain 30% B; 2–10 min, 30–0% B; 10–12 min, 0–30% B. The sample injection volume was set at 1 μL for the separation of the different components, column temperature was 40 °C. Q-TOF-MS/MS analysis was performed on a Triple TOF 5600*^plus^* Mass spectrometer (AB SCIEX, Framingham, MA, USA) equipped with an ESI source, which was run in the negative ionization mode. The scan range was set at 100–2000 Da. The system was run with the following parameters: ion spray voltage, −4500 V; ion source heater, 550 °C; curtain gas (CUR, N_2_), 30 psi; nebulizing gas (GS1, Air), 50 psi; Tis gas (GS2, Air), 50 psi. The declustering potential (DP) was set at −100 V, whereas the collision energy (CE) was set at −40 V with a collision energy spread (CES) of 20 V. The RAF products were quantified by extracting the corresponding ion formula (e.g., [C_37_H_29_N_5_O_10_-H]^−^ for moracin C-DPPH•) from the total ion chromatogram and integrating the corresponding peak. 

### 3.6. Statistical Analysis

Each experiment was performed in triplicate and the data were recorded as mean ± SD (standard deviation). The dose–response curves were plotted using Origin 6.0 professional software (OriginLab, Northampton, MA, USA). The IC_50_ value was defined as the final concentration of 50% radical inhibition (or relative reducing power). It was calculated by linear regression analysis, and expressed as the mean ± SD (*n* = 3). The linear regression was analyzed using Origin 6.0. Determination of significant differences between the mean IC_50_ values was performed using one-way ANOVA and the *t*-test. The analysis was performed using SPSS software 13.0 (SPSS Inc., Chicago, IL, USA) for windows. *p* < 0.05 was considered to be statistically significant.

## 4. Conclusions

Both moracin C and *iso*-moracin C can inhibit ROS, likely through redox-related pathways (specially ET and H^+^-transfer) and a non-redox-related RAF pathway. In the redox-related pathways, a double bond at the conjugation position can enhance the ET and H^+^-transfer potential. However, in the non-redox-related pathway, the double bond position hardly affected the RAF potential.

## Supplementary Materials

The following supplementary materials are available online. Video S1 and S2: Animations of moracin C and *iso*-moracin C; Figure S1: Structure of artoindonesianin B; Figure S2–S5: Dose response curves; Figure S6: Photos of moracin C and *iso*-moracin C.

## Abbreviations

The following abbreviations are used in this manuscript:

ABTS	2,2′-azino-bis (3-ethylbenzo-thiazoline-6-sulfonic acid) diammonium salt
DPPH•	1,1-diphenyl-2-picryl-hydrazl
ET	electron-transfer
FBS	fetal bovine serum
FRAP	ferric reducing antioxidant power
HAT	hydrogen atom transfer
ROS	reactive oxygen species
RAF	radical-adduct-formation
SD	standard deviation
TPTZ	2,4,6-tris(2-pyridyl-s-triazine)
Trolox	(±)-6-hydroxyl-2,5,7,8-tetramethlychromane-2-carboxylic acid
