# Supplementary material for: Effect of Double Bond Position on 2-Phenyl-benzofuran Antioxidants: A Comparative Study of Moracin C and Iso-Moracin C
Source: Molecules. 2018 Mar 24;23(4):754. doi: 10.3390/molecules23040754 (PMC6017532; doi:10.3390/molecules23040754)
Supplement: Supplementary file 1 [file molecules-23-00754-s001.zip › Suppls/Figure S2-S5 Dose response curves.docx]

**Figure S2-S5**

**Double Bond Position Effect on 2-Phenyl-benzofuran Antioxidants: A Comparative Study of Moracin C and *Iso*-moracin C**

**Xican Li ^1, 2,†, *^, Hong Xie ^1, 2,†^**, **Ruicai Zhan ^1^, and Dongfeng Chen ^3, 4, *^**

^1^ School of Chinese Herbal Medicine; xiehongxh1@163.com (H.X.); [1664832623@qq.com (R.Z.);](mailto:1664832623@qq.com%20(R.Z.);)

^2^ Innovative Research & Development Laboratory of TCM;

^3^ School of Basic Medical Science;

^4^ The Research Center of Basic Integrative Medicine, Guangzhou University of Chinese Medicine, Waihuan East Road No. 232, Guangzhou Higher Education Mega Center, Guangzhou 510006, China

^*^ Corresponding author. E-mail: [lixican@126. com](mailto:lixican@126.com)(X.L.); chen888@gzucm.edu.cn; Tel.: +86-203-935-8076

**^†^**These authors contributed equally to this work.


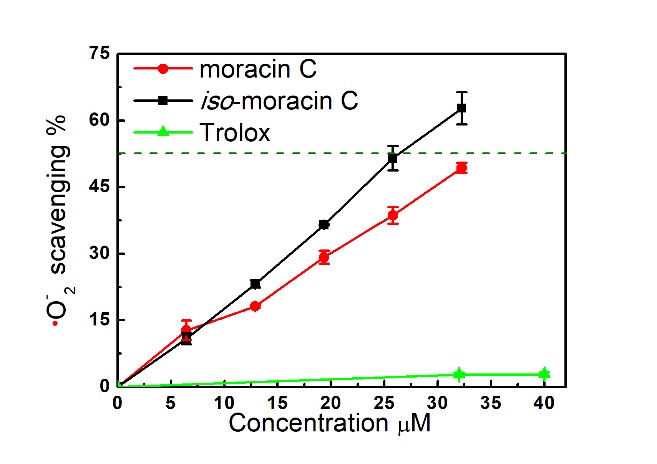


Figure S2**:** The dose response curves of moracin C and iso-moracin C in •O_2_^-^-inhibition assay. Each value is expressed as mean ± SD (n = 3).


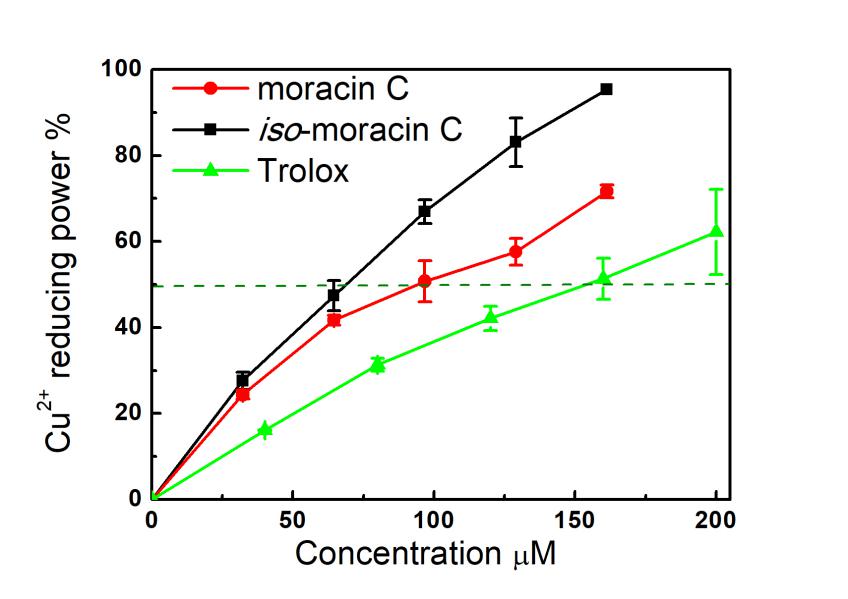


Figure S3**:** The dose response curves of moracin C and iso-moracin C in Cu^2+^-reducing power assay (CUPRAC). Each value is expressed as mean ± SD (n = 3).


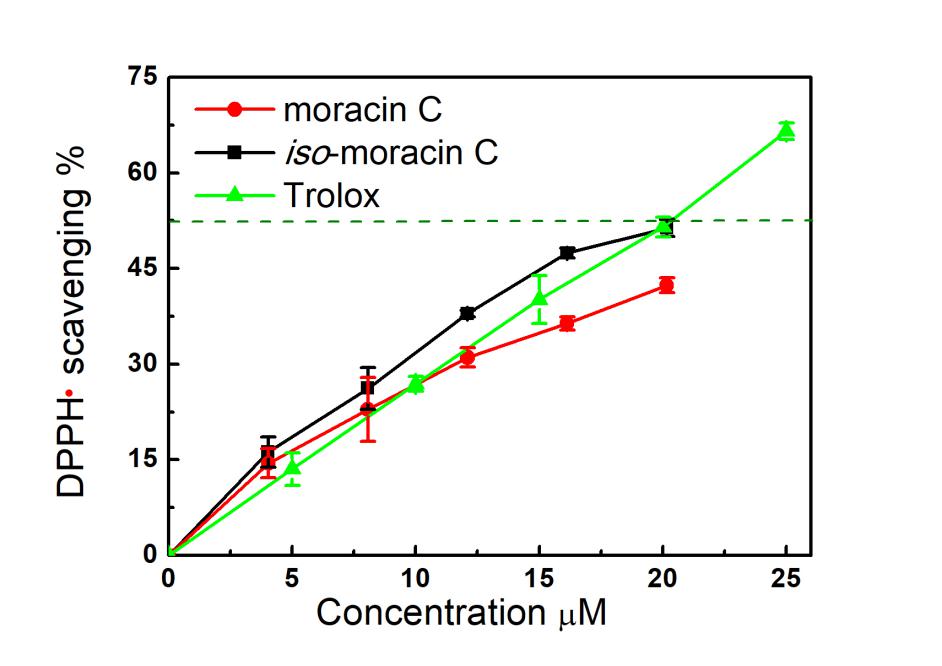


Figure S4**:** The dose response curves of moracin C and iso-moracin C in DPPH•-scavenging assay. Each value is expressed as mean ± SD (n = 3).


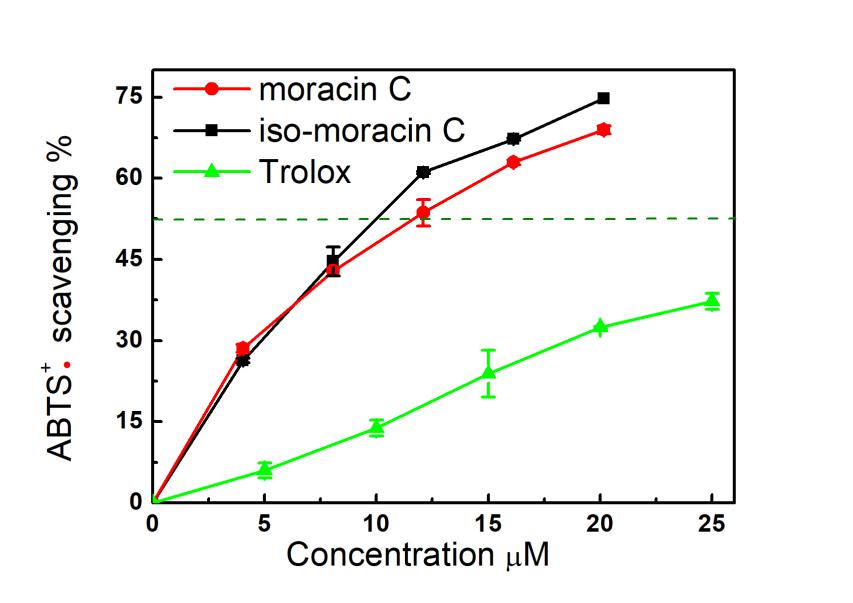


Figure S5**:** The dose response curves of moracin C and iso-moracin C in ABTS^+^•-scavenging assay. Each value is expressed as mean ± SD (n = 3).
