# Supplementary figures and images for: Effect of Double Bond Position on 2-Phenyl-benzofuran Antioxidants: A Comparative Study of Moracin C and Iso-Moracin C
Source: Molecules. 2018 Mar 24;23(4):754. doi: 10.3390/molecules23040754 (PMC6017532; doi:10.3390/molecules23040754)

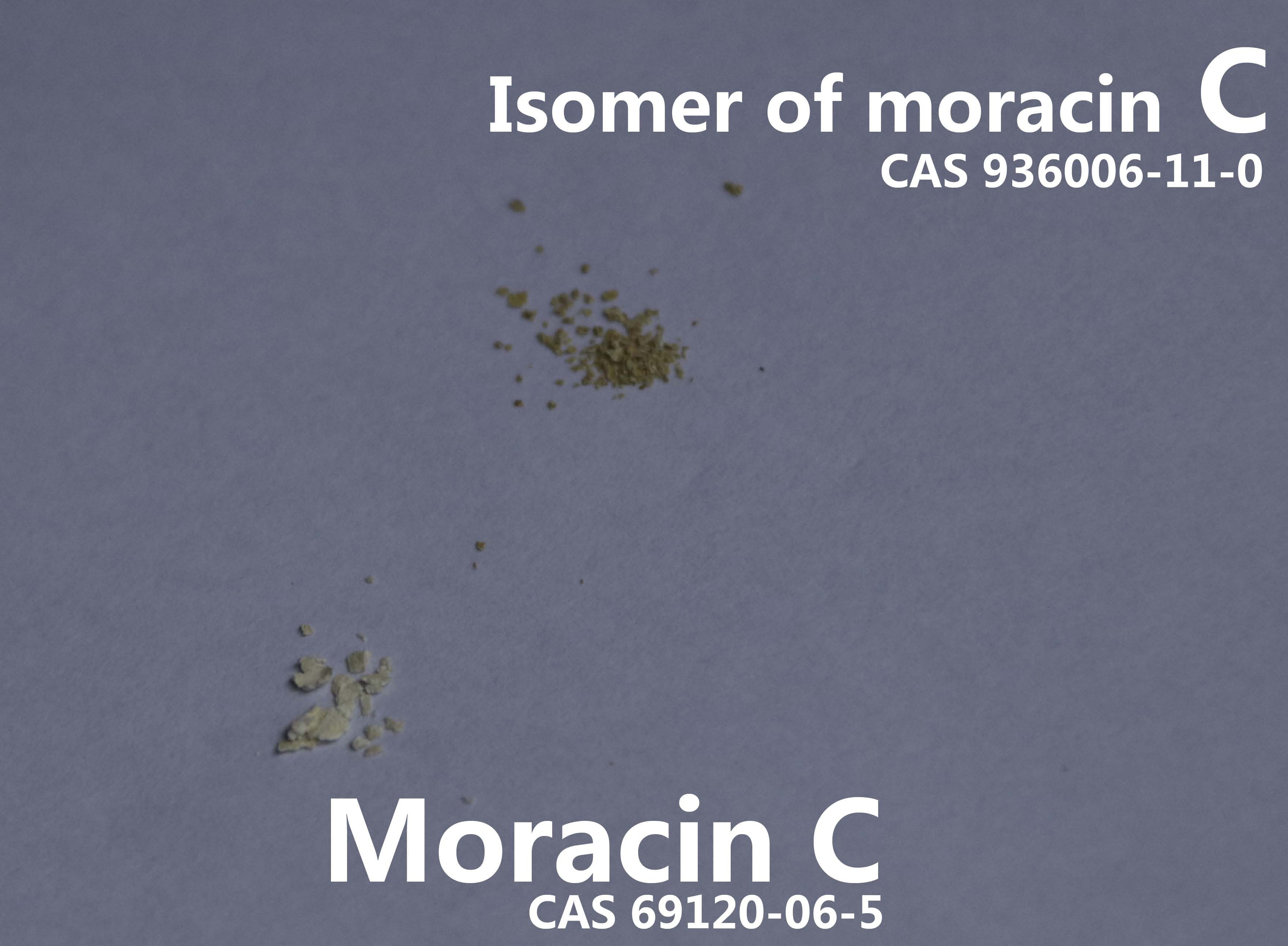

Supplement: Supplementary file 1 [file molecules-23-00754-s001.zip › Suppls/Figure S6 Photoes.jpg]
